# 1-(2-Hy­droxy-4,5-dimeth­oxy­phen­yl)ethanone

**DOI:** 10.1107/S1600536812051057

**Published:** 2012-12-22

**Authors:** Stefania M. Scalzullo, Sanaz Khorasani, Joseph P. Michael

**Affiliations:** a Molecular Sciences Institute, School of Chemistry, University of the Witwatersrand, PO Wits 2050, Johannesburg, South Africa

## Abstract

The mol­ecular structure of the title compound, C_10_H_12_O_4_, contains an intra­molecular hydrogen bond between the phenol and acetyl substituents. In the crystal, C—H⋯π inter­actions act between the mol­ecules in a cyclic manner to stabilize stacks of mol­ecules along the *b* axis. Several C—H⋯O inter­actions are present between the stacks.

## Related literature

For a review on lamellarin alkaloids, see: Fan *et al.* (2008 ▶). The experimental procedure of Combes *et al.* (2002 ▶) for a related Fries rearrangement was adapted for the synthesis of the title compound. For alternative syntheses of the title compound by Fries rearrangement, see: Ploypradith *et al.* (2003 ▶); Nolan *et al.* (2009 ▶).
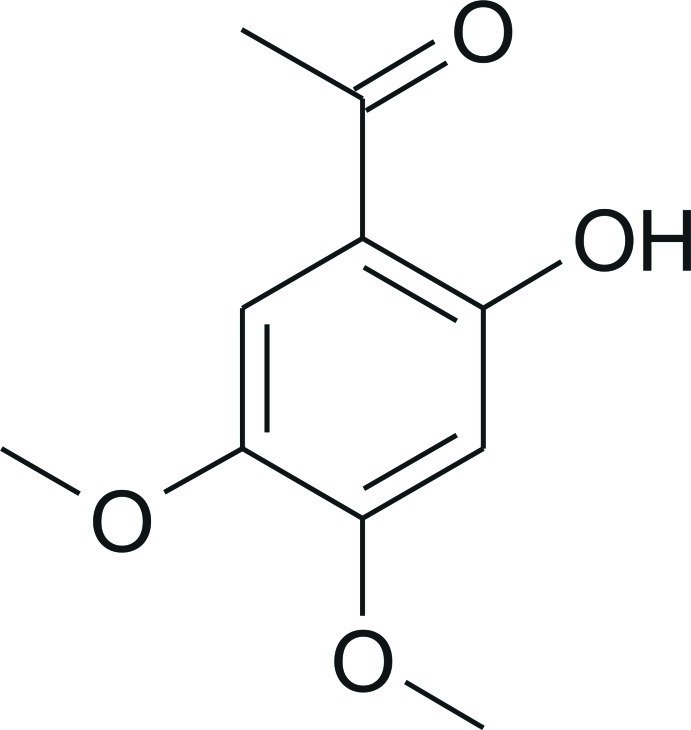


## Experimental

### 

#### Crystal data

C_10_H_12_O_4_
*M* *_r_* = 196.20
Orthorhombic, 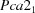

*a* = 19.1740 (12) Å
*b* = 5.5026 (3) Å
*c* = 8.9956 (5) Å
*V* = 949.10 (9) Å^3^
*Z* = 4
Mo *K*α radiation
μ = 0.11 mm^−1^
*T* = 173 K
0.41 × 0.32 × 0.20 mm

#### Data collection

Bruker APEXII CCD diffractometer
4718 measured reflections
1214 independent reflections
1106 reflections with *I* > 2σ(*I*)
*R*_int_ = 0.032

#### Refinement

*R*[*F* ^2^ > 2σ(*F* ^2^)] = 0.032
*wR*(*F* ^2^) = 0.085
*S* = 1.06
1214 reflections
134 parameters
1 restraint
H atoms treated by a mixture of independent and constrained refinement
Δρ_max_ = 0.20 e Å^−3^
Δρ_min_ = −0.18 e Å^−3^

### 

Data collection: *APEX2* (Bruker, 2005 ▶); cell refinement: *SAINT* (Bruker, 2005 ▶); data reduction: *SAINT*; program(s) used to solve structure: *SHELXS97* (Sheldrick, 2008 ▶); program(s) used to refine structure: *SHELXL97* (Sheldrick, 2008 ▶); molecular graphics: *ORTEP-3 for Windows* (Farrugia, 2012 ▶) and *SCHAKAL99* (Keller, 1999 ▶); software used to prepare material for publication: *WinGX* (Farrugia, 2012 ▶) and *PLATON* (Spek, 2009 ▶).

## Supplementary Material

Crystal structure: contains datablock(s) global, I. DOI: 10.1107/S1600536812051057/nk2196sup1.cif

Structure factors: contains datablock(s) I. DOI: 10.1107/S1600536812051057/nk2196Isup2.hkl

Supplementary material file. DOI: 10.1107/S1600536812051057/nk2196Isup3.cml

Additional supplementary materials:  crystallographic information; 3D view; checkCIF report

## Figures and Tables

**Table 1:** Hydrogen-bond geometry (Å, °) *Cg*1 is the centroid of the C1–C6 ring.

*D*—H⋯*A*	*D*—H	H⋯*A*	*D*⋯*A*	*D*—H⋯*A*
O1—H1⋯O4	0.93 (3)	1.71 (3)	2.549 (2)	150 (2)
C8—H8*C*⋯O3^i^	0.98	2.40	3.365 (2)	168
C9—H9*B*⋯O3^ii^	0.98	2.57	3.513 (3)	162
C10—H10*C*⋯O2^i^	0.98	2.53	3.334 (3)	139
C8—H8*B*⋯*Cg*1^iii^	0.98	2.80	3.738 (3)	160
C9—H9*A*⋯*Cg*1^iv^	0.98	2.90	3.828 (3)	158

Symmetry codes: (i) 
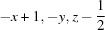
; (ii) 
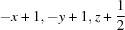
; (iii) 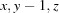; (iv) 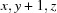.

**Fig. 1.**
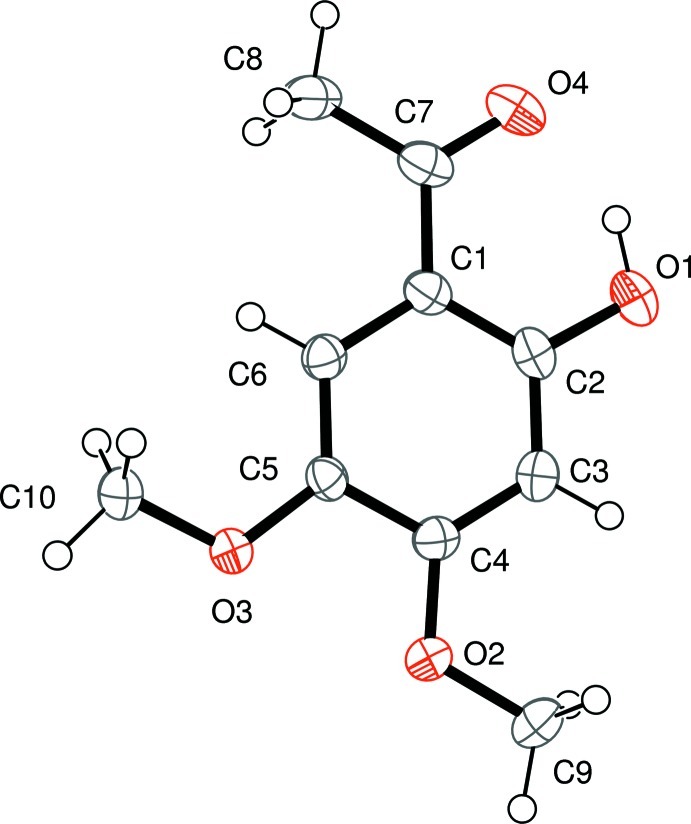
The molecular structure of (I), showing the atomic numbering scheme. Displacement ellipsoids are drawn at the 50% probability level.

**Fig. 2.**
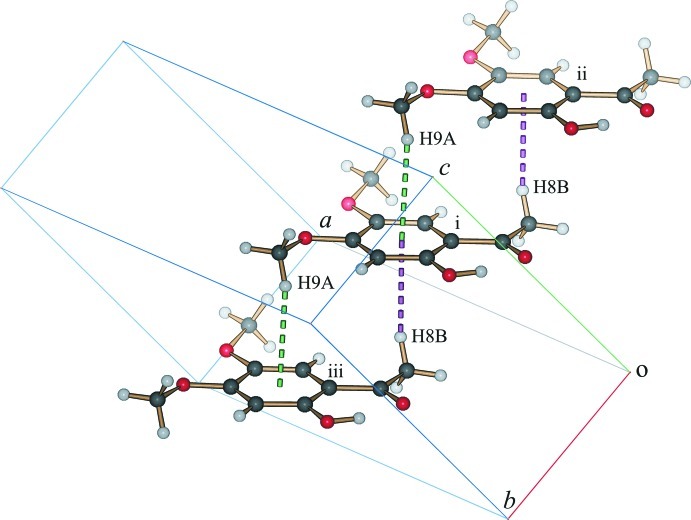
C-H···π interactions in the structure of (I) stabilising stacks of molecules along the b axis. [Symmetry codes: (i) x, y, z; (ii) x, -1+y, z; (iii) x, 1+y, z.]

**Fig. 3.**
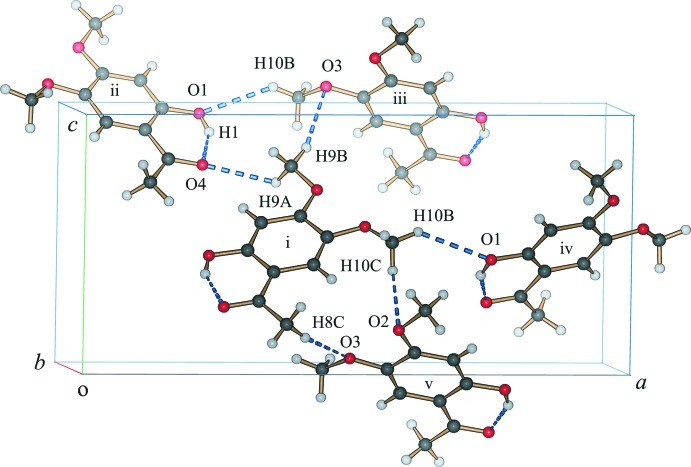
C-H···O interactions between the stacks of molecules in the structure of (I). [Symmetry codes: (i) x, y, z; (ii) 1/2-x, 1+y, 1/2+z; (iii) 1-x, 1-y, 1/2+z; (iv) 1/2+x, -y, z; (v) 1-x, -y, -1/2+z.]

## supplementary crystallographic information

### Comment

The title organic compound (Fig. 1), required as an intermediate in the synthesis of lamellarin alkaloids (Fan *et al.*, 2008), was prepared by Fries rearrangement of 3,4-dimethoxyphenyl acetate with boron trifluoride etherate. Syntheses of the same compound by related Fries rearrangements have been reported by Ploypradith *et al.* (2003) and Nolan *et al.* (2009).

The compound crystallizes in the polar space group *Pca*2_1_. An intramolecular hydrogen bond exists between the phenol and acetyl groups (Fig. 1). Molecules related by translation along the *b* axis interact through two C—H···π interactions which act between the molecules in a cyclic manner. These stabilize stacks of molecules along the *b* axis (Fig. 2). Several C—H···O interactions exist in the structure, the most significant being listed in Table 1. These act between the stacks to stabilize the structure (Fig. 3).

### Experimental

The experimental procedure of Combes *et al.*. (2002) for a related Fries rearrangement was adapted for the synthesis of the title compound. Boron trifloride etherate (20 ml, 23.1 g, 163 mmol) was cautiously added to ice-cooled 3,4-dimethoxyphenyl acetate (7.99 g, 40.7 mmol). The mixture was warmed to room temperature, then heated to 383 K for 5 h before being cooled again to room temperature and stirred for an additional 18 h. Water (50 ml) was added, resulting in the precipitation of a brown solid. This was filtered off, washed with a copious amount of water, then recrystallized from methanol to afford 1-(2-hydroxy-4,5-dimethoxyphenyl)ethanone (4.90 g, 61%) as dark yellow blocks, m.p. 385–386 K.

### Refinement

All H atoms attached to carbon were positioned geometrically, and allowed to ride on their parent atoms, with C—H bond lengths of 0.95 Å (CH) or 0.98 Å (CH_3_), and isotropic displacement parameters set to 1.2 (CH) or 1.5 times (CH_3_) the *U*_eq_ of the parent atom. Friedel pairs were merged during final refinement.

### Crystal data

**Table:**

C_10_H_12_O_4_	*F*(000) = 416
*M**_r_* = 196.20	*D*_x_ = 1.373 Mg m^−^^3^
Orthorhombic, *P**c**a*2_1_	Mo *K*α radiation, λ = 0.71073 Å
Hall symbol: P 2c -2ac	Cell parameters from 2177 reflections
*a* = 19.1740 (12) Å	θ = 3.1–27.7°
*b* = 5.5026 (3) Å	µ = 0.11 mm^−^^1^
*c* = 8.9956 (5) Å	*T* = 173 K
*V* = 949.10 (9) Å^3^	Block, colourless
*Z* = 4	0.41 × 0.32 × 0.20 mm

### Data collection

**Table:**

Bruker APEXII CCD diffractometer	1106 reflections with *I* > 2σ(*I*)
Radiation source: fine-focus sealed tube	*R*_int_ = 0.032
Graphite monochromator	θ_max_ = 28.0°, θ_min_ = 2.1°
φ and ω scans	*h* = −25→24
4718 measured reflections	*k* = −7→7
1214 independent reflections	*l* = −11→10

### Refinement

**Table:**

Refinement on *F*^2^	Primary atom site location: structure-invariant direct methods
Least-squares matrix: full	Secondary atom site location: difference Fourier map
*R*[*F*^2^ > 2σ(*F*^2^)] = 0.032	Hydrogen site location: inferred from neighbouring sites
*wR*(*F*^2^) = 0.085	H atoms treated by a mixture of independent and constrained refinement
*S* = 1.06	*w* = 1/[σ^2^(*F*_o_^2^) + (0.0504*P*)^2^ + 0.0546*P*] where *P* = (*F*_o_^2^ + 2*F*_c_^2^)/3
1214 reflections	(Δ/σ)_max_ < 0.001
134 parameters	Δρ_max_ = 0.20 e Å^−^^3^
1 restraint	Δρ_min_ = −0.18 e Å^−^^3^

### Special details

**Table:**

Geometry. All e.s.d.'s (except the e.s.d. in the dihedral angle between two l.s. planes) are estimated using the full covariance matrix. The cell e.s.d.'s are taken into account individually in the estimation of e.s.d.'s in distances, angles and torsion angles; correlations between e.s.d.'s in cell parameters are only used when they are defined by crystal symmetry. An approximate (isotropic) treatment of cell e.s.d.'s is used for estimating e.s.d.'s involving l.s. planes.
Refinement. Refinement of *F*^2^ against ALL reflections. The weighted *R*-factor *wR* and goodness of fit *S* are based on *F*^2^, conventional *R*-factors *R* are based on *F*, with *F* set to zero for negative *F*^2^. The threshold expression of *F*^2^ > σ(*F*^2^) is used only for calculating *R*-factors(gt) *etc*. and is not relevant to the choice of reflections for refinement. *R*-factors based on *F*^2^ are statistically about twice as large as those based on *F*, and *R*- factors based on ALL data will be even larger.

### Fractional atomic coordinates and isotropic or equivalent isotropic displacement parameters (Å^2^)

**Table:**

	*x*	*y*	*z*	*U*_iso_*/*U*_eq_	
C1	0.34395 (9)	−0.0487 (3)	0.3973 (2)	0.0287 (4)	
C2	0.30480 (9)	0.1288 (3)	0.4721 (2)	0.0322 (4)	
C3	0.33683 (9)	0.2885 (3)	0.5713 (2)	0.0307 (4)	
H3	0.3100	0.4093	0.6205	0.037*	
C4	0.40755 (9)	0.2705 (3)	0.59765 (19)	0.0262 (4)	
C5	0.44769 (8)	0.0884 (3)	0.5258 (2)	0.0248 (4)	
C6	0.41609 (9)	−0.0644 (3)	0.4263 (2)	0.0271 (4)	
H6	0.4432	−0.1829	0.3758	0.033*	
C7	0.30942 (10)	−0.2140 (4)	0.2923 (2)	0.0346 (4)	
C8	0.35122 (11)	−0.4047 (4)	0.2122 (3)	0.0395 (5)	
H8A	0.3210	−0.4921	0.1425	0.059*	
H8B	0.3706	−0.5194	0.2845	0.059*	
H8C	0.3893	−0.3269	0.1573	0.059*	
C9	0.40535 (10)	0.5915 (3)	0.7748 (3)	0.0357 (4)	
H9A	0.3838	0.7080	0.7064	0.053*	
H9B	0.4368	0.6778	0.8426	0.053*	
H9C	0.3690	0.5091	0.8323	0.053*	
C10	0.55610 (10)	−0.1202 (3)	0.5150 (3)	0.0364 (5)	
H10A	0.5342	−0.2712	0.5491	0.055*	
H10B	0.6036	−0.1097	0.5548	0.055*	
H10C	0.5578	−0.1188	0.4061	0.055*	
O1	0.23555 (7)	0.1547 (3)	0.4504 (2)	0.0469 (4)	
O2	0.44429 (7)	0.4153 (2)	0.69134 (15)	0.0308 (3)	
O3	0.51633 (6)	0.0824 (3)	0.56605 (17)	0.0321 (3)	
O4	0.24611 (9)	−0.2002 (3)	0.2697 (2)	0.0501 (4)	
H1	0.2231 (13)	0.032 (5)	0.385 (4)	0.061 (8)*	

### Atomic displacement parameters (Å^2^)

**Table:**

	*U*^11^	*U*^22^	*U*^33^	*U*^12^	*U*^13^	*U*^23^
C1	0.0269 (9)	0.0322 (9)	0.0270 (9)	−0.0045 (7)	−0.0010 (7)	0.0009 (8)
C2	0.0227 (8)	0.0382 (10)	0.0357 (10)	−0.0005 (8)	0.0018 (8)	0.0028 (9)
C3	0.0280 (8)	0.0334 (9)	0.0308 (10)	0.0027 (7)	0.0050 (8)	0.0000 (8)
C4	0.0297 (9)	0.0267 (8)	0.0222 (8)	−0.0035 (7)	0.0024 (7)	0.0010 (7)
C5	0.0214 (8)	0.0284 (8)	0.0247 (8)	−0.0017 (7)	0.0017 (7)	0.0017 (7)
C6	0.0278 (8)	0.0279 (8)	0.0257 (9)	−0.0015 (7)	0.0036 (7)	−0.0002 (7)
C7	0.0331 (10)	0.0384 (10)	0.0322 (10)	−0.0113 (8)	−0.0023 (8)	0.0015 (9)
C8	0.0439 (12)	0.0401 (11)	0.0345 (10)	−0.0141 (9)	0.0022 (9)	−0.0072 (9)
C9	0.0424 (11)	0.0304 (9)	0.0342 (9)	0.0044 (7)	0.0023 (9)	−0.0072 (8)
C10	0.0279 (8)	0.0346 (9)	0.0468 (12)	0.0064 (8)	−0.0022 (9)	−0.0067 (9)
O1	0.0229 (7)	0.0571 (9)	0.0608 (11)	0.0031 (7)	−0.0054 (7)	−0.0103 (9)
O2	0.0298 (7)	0.0323 (6)	0.0304 (7)	0.0003 (5)	−0.0003 (5)	−0.0072 (6)
O3	0.0235 (6)	0.0356 (6)	0.0371 (7)	0.0029 (5)	−0.0026 (5)	−0.0097 (6)
O4	0.0327 (7)	0.0585 (9)	0.0589 (10)	−0.0089 (7)	−0.0106 (8)	−0.0106 (9)

### Geometric parameters (Å, º)

**Table:**

C1—C2	1.403 (3)	C7—C8	1.504 (3)
C1—C6	1.410 (2)	C8—H8A	0.9800
C1—C7	1.469 (3)	C8—H8B	0.9800
C2—O1	1.349 (2)	C8—H8C	0.9800
C2—C3	1.395 (3)	C9—O2	1.436 (2)
C3—C4	1.380 (2)	C9—H9A	0.9800
C3—H3	0.9500	C9—H9B	0.9800
C4—O2	1.357 (2)	C9—H9C	0.9800
C4—C5	1.419 (2)	C10—O3	1.426 (2)
C5—O3	1.365 (2)	C10—H10A	0.9800
C5—C6	1.370 (2)	C10—H10B	0.9800
C6—H6	0.9500	C10—H10C	0.9800
C7—O4	1.233 (2)	O1—H1	0.93 (3)
			
C2—C1—C6	118.60 (16)	C7—C8—H8A	109.5
C2—C1—C7	119.87 (16)	C7—C8—H8B	109.5
C6—C1—C7	121.53 (16)	H8A—C8—H8B	109.5
O1—C2—C3	117.31 (18)	C7—C8—H8C	109.5
O1—C2—C1	122.06 (18)	H8A—C8—H8C	109.5
C3—C2—C1	120.63 (15)	H8B—C8—H8C	109.5
C4—C3—C2	119.84 (16)	O2—C9—H9A	109.5
C4—C3—H3	120.1	O2—C9—H9B	109.5
C2—C3—H3	120.1	H9A—C9—H9B	109.5
O2—C4—C3	125.10 (16)	O2—C9—H9C	109.5
O2—C4—C5	114.56 (15)	H9A—C9—H9C	109.5
C3—C4—C5	120.33 (16)	H9B—C9—H9C	109.5
O3—C5—C6	125.79 (15)	O3—C10—H10A	109.5
O3—C5—C4	114.79 (15)	O3—C10—H10B	109.5
C6—C5—C4	119.42 (15)	H10A—C10—H10B	109.5
C5—C6—C1	121.15 (15)	O3—C10—H10C	109.5
C5—C6—H6	119.4	H10A—C10—H10C	109.5
C1—C6—H6	119.4	H10B—C10—H10C	109.5
O4—C7—C1	120.76 (19)	C2—O1—H1	105.6 (16)
O4—C7—C8	119.24 (18)	C4—O2—C9	116.82 (14)
C1—C7—C8	120.00 (17)	C5—O3—C10	116.68 (14)
			
C6—C1—C2—O1	−179.90 (19)	O3—C5—C6—C1	177.32 (16)
C7—C1—C2—O1	−0.3 (3)	C4—C5—C6—C1	−2.0 (2)
C6—C1—C2—C3	0.9 (3)	C2—C1—C6—C5	0.5 (2)
C7—C1—C2—C3	−179.47 (17)	C7—C1—C6—C5	−179.13 (17)
O1—C2—C3—C4	−179.95 (18)	C2—C1—C7—O4	−0.6 (3)
C1—C2—C3—C4	−0.7 (3)	C6—C1—C7—O4	178.99 (19)
C2—C3—C4—O2	179.97 (17)	C2—C1—C7—C8	−179.90 (17)
C2—C3—C4—C5	−0.8 (3)	C6—C1—C7—C8	−0.3 (3)
O2—C4—C5—O3	2.1 (2)	C3—C4—O2—C9	3.5 (3)
C3—C4—C5—O3	−177.21 (16)	C5—C4—O2—C9	−175.75 (15)
O2—C4—C5—C6	−178.53 (15)	C6—C5—O3—C10	−8.9 (3)
C3—C4—C5—C6	2.2 (2)	C4—C5—O3—C10	170.47 (16)

### Hydrogen-bond geometry (Å, º)

Cg1 is the centroid of the C1–C6 ring.

**Table:**

*D*—H···*A*	*D*—H	H···*A*	*D*···*A*	*D*—H···*A*
O1—H1···O4	0.93 (3)	1.71 (3)	2.549 (2)	150 (2)
C8—H8*C*···O3^i^	0.98	2.40	3.365 (2)	168
C9—H9*B*···O3^ii^	0.98	2.57	3.513 (3)	162
C10—H10*C*···O2^i^	0.98	2.53	3.334 (3)	139
C8—H8*B*···*Cg*1^iii^	0.98	2.80	3.738 (3)	160
C9—H9*A*···*Cg*1^iv^	0.98	2.90	3.828 (3)	158

Symmetry codes: (i) −*x*+1, −*y*, *z*−1/2; (ii) −*x*+1, −*y*+1, *z*+1/2; (iii) *x*, *y*−1, *z*; (iv) *x*, *y*+1, *z*.

## References

[bb1] Bruker (2005). *APEX2* and *SAINT* Bruker AXS Inc., Madison, Wisconsin, USA.

[bb2] Combes, S., Finet, J.-P. & Siri, D. (2002). *J. Chem. Soc. Perkin Trans. 1*, pp. 38–44.

[bb3] Fan, H., Peng, J., Hamann, M. T. & Hu, J.-F. (2008). *Chem. Rev.* **108**, 264–287. 10.1021/cr078199m PMC4928200 18095718

[bb4] Farrugia, L. J. (2012). *J. Appl. Cryst.* **45**, 849–854.

[bb5] Keller, E. (1999). *SCHAKAL99* University of Freiberg, Germany.

[bb6] Nolan, K. A., Doncaster, J. R., Dunstan, M. S., Scott, K. A., Frenkel, A. D., Siegel, D., Ross, D., Barnes, J., Levy, C., Leys, D., Whitehead, R. C., Stratford, I. J. & Bryce, R. A. (2009). *J. Med. Chem.* **52**, 7142–7156. 10.1021/jm9011609 19877692

[bb7] Ploypradith, P., Jinaglueng, W., Pavaro, C. & Ruchirawal, S. (2003). *Tetrahedron Lett.* **44**, 1363–1366.

[bb8] Sheldrick, G. M. (2008). *Acta Cryst.* A**64**, 112–122. 10.1107/S0108767307043930 18156677

[bb9] Spek, A. L. (2009). *Acta Cryst.* D**65**, 148–155. 10.1107/S090744490804362X PMC2631630 19171970

